# Intratumoral and peritumoral radiomics based on automated breast volume scanner for predicting human epidermal growth factor receptor 2 status

**DOI:** 10.3389/fonc.2025.1556317

**Published:** 2025-04-16

**Authors:** Hao Zhang, Qing Miao, Yan Fu, Ruike Pan, Qing Jin, Changjiang Gu, Xuejun Ni

**Affiliations:** ^1^ From the Department of Medical Ultrasound, Affiliated Hospital of Nantong University, Nantong, China; ^2^ From the Department of Ultrasound, Jiangsu Cancer Hospital, Nanjing, China; ^3^ From the Department of Ultrasound, The First People’s Hospital of Lianyungang, Lianyungang, China; ^4^ From the Department of Ultrasound, Kunshan Traditional Chinese Medicine Hospital, Kunshan, China; ^5^ From the Department of General Surgery, Affiliated Hospital of Nantong University, Nantong, China

**Keywords:** automated breast volume scanner, radiomics, peritumoral, human epidermal growth factor receptor 2, breast cancer

## Abstract

**Purpose:**

To develop an intratumoral and peritumoral radiomics model using Automated Breast Volume Scanner (ABVS) for noninvasive preoperative prediction of Human Epidermal Growth Factor Receptor 2 (HER2) status.

**Methods:**

This retrospective study analyzed 384 lesions from 379 patients with pathologically confirmed breast cancer across four hospitals. Two tasks were defined: Task 1 to distinguish HER2-negative from HER2-positive cases and Task 2 to differentiate HER2-zero from HER2-low status. For each classification task, three models were built: Model 1 included radiomics features from the tumor region alone; Model 2 included features from both the tumor region and a 5mm peritumoral region; and Model 3 incorporated features from the tumor region, the 5mm peritumoral region, and the 5-10mm peritumoral region. The performance of the model was evaluated using receiver operating characteristic (ROC) curves, with key metrics including the area under the curve (AUC), sensitivity, specificity, and accuracy.

**Results:**

In the classification tasks, Model 2 demonstrated superior predictive performance across multiple datasets. For Task 1, it achieved the highest AUC (0.844), exceptional sensitivity (0.955), and satisfactory accuracy (0.787) in the validation set, and outperformed other models in the test set with an AUC of 0.749 and sensitivity of 0.885, highlighting its robustness and clinical applicability. For Task 2, Model 2 exhibited the highest AUC (0.801), sensitivity (0.862), and accuracy (0.808) in the test set, with consistent performance across the training (AUC 0.850) and validation sets (AUC 0.801). Model 3, which combines intratumoral and peritumoral features, did not demonstrate significant improvements over the intratumoral-only model in the two classification tasks. These results underscore the value of incorporating peritumoral radiomics features, particularly within a 5mm margin, to enhance predictive performance compared to intratumoral-only models.

**Conclusion:**

The radiomics model integrating intratumoral and appropriate peritumoral features significantly outperformed the model based on intratumoral features alone. This integrated approach holds strong potential for noninvasive, preoperative prediction of HER2 status.

## 1 Introduction

Breast cancer remains the most prevalent malignancy in women, accounting for 32% of new cancer diagnoses and 15% of cancer-related mortality among women (1, 2). Its molecular heterogeneity necessitates precise subtyping based on immunohistochemical (IHC) markers, including estrogen receptor (ER), progesterone receptor (PR), HER2, Ki-67, and Fluorescence *In Situ* Hybridization (FISH), with HER2 positivity (15%-20% cases) correlating with aggressive phenotypes and poor prognosis (3–6). Emerging evidence redefines HER2-low expression (IHC 1+ or 2+/FISH-) as a distinct entity, showing differential responses to novel antibody-drug conjugates (ADCs) like trastuzumab deruxtecan (T-DXd). Compared to HER2-zero tumors, HER2-low subtypes exhibit distinct mutation profiles (higher PIK3CA, lower TP53) and improved survival with ADCs therapies (7–10). This paradigm shift underscores the urgent need for noninvasive methods to accurately stratify HER2 status, particularly the critical differentiation between HER2-low and HER2-zero within traditionally “HER2-negative” cohorts.

Radiomics is a cutting-edge approach in medical imaging, employs high-throughput feature extraction to decode lesion pathophysiology (11, 12). It overcomes traditional visual assessment limitations by quantifying tumor heterogeneity across morphological, textural, and functional metabolic dimensions. Unlike the localized sampling of needle biopsies, radiomics noninvasively analyzes spatial heterogeneity across entire lesions and their microenvironments (13). Current radiomics approaches using magnetic resonance imaging (MRI) have demonstrated success in HER2 evaluation through quantitative analysis of tumor heterogeneity (14, 15). Despite ultrasound radiomics identified a correlation between regional entropy in radiomics features and the presence of calcifications in HER2-positive breast cancer ultrasound images (16), faces standardization challenges due to operator-dependent variability in image acquisition. ABVS addresses this limitation through standardized 3D imaging and unique coronal plane visualization (17, 18), its applications remain confined to HER2-positive and negative classification, axillary lymph node metastasis (ALNM), and KI67 expression levels (19–21). Notably, existing HER2-low investigations exclusively rely on MRI (14, 22, 23), ignoring the potential of ultrasound-based radiomics. Furthermore, conventional radiomics models focus predominantly on intratumoral features, neglecting the biological significance of peritumoral regions where immune microenvironment alterations and tumor-stroma interactions occur (24–26).

To bridge these gaps, we propose a dual-region ABVS radiomics strategy integrating both intratumoral and peritumoral signatures. Our approach leverages three key innovations: First, ABVS’s operator-independent acquisition standardizes radiomics feature extraction, overcoming conventional ultrasound’s reproducibility limitations. Second, the coronal plane visualization enables 3D characterization of tumor-microenvironment interactions, capturing spatial heterogeneity patterns potentially linked to HER2 expression biology. Third, by incorporating peritumoral features reflecting immune landscape changes and stromal activation, our model extends beyond traditional tumor-centric analyses. We hypothesize that this multimodal integration will improve HER2-low detection accuracy compared to MRI-based or intratumoral only models.

This study aims to develop and validate the first ABVS radiomics model for preoperative HER2 status prediction, with specific emphasis on distinguishing HER2-low from HER2-zero expression. By establishing a noninvasive imaging biomarker system that combines tumor intrinsic features with microenvironmental signatures, our work enables early identification of HER2-low patients who may benefit from emerging ADCs therapies.

## 2 Materials and methods

### 2.1 Patient population

This retrospective multicenter study was approved by the institutional review boards of all participating institutions, with a waiver of written informed consent from the patients. The study enrolled breast cancer patients from several institutions, including Hospital 1 (between November 2019 and December 2023), Hospital 2 (from November 2021 to March 2023), Hospital 3 (from June 2020 to August 2023), and Hospital 4 (from August 2017 to March 2023). All patients underwent preoperative ABVS and received pathological confirmation of cancer following surgery. The original ABVS images were stored on the workstation, and images in DICOM format were imported into specialized software to ensure the integrity and quality of the initial imaging data.

Inclusion Criteria (1): Patients who underwent surgical excision of the target tumor, with only lesions having corresponding pathological analysis included in cases of multiple lesions. (2) Pathologically confirmed primary breast cancer, with available IHC markers for ER, PR, Ki67, and HER2 status. (3) Preoperative ABVS performed within two weeks prior to surgery. (4) Patients who had not received neoadjuvant chemotherapy or other treatments before the ABVS examination.

Exclusion Criteria: (1) Any preoperative interventions or treatments (e.g., radiotherapy, chemotherapy, radiofrequency ablation, biopsy) prior to ABVS. (2) Target lesions that are unclear on ABVS images or lack a visible region of interest (ROI) due to artifacts. (3) Cases where pathological and IHC data were insufficient for molecular subtype classification.

**Figure 1:**
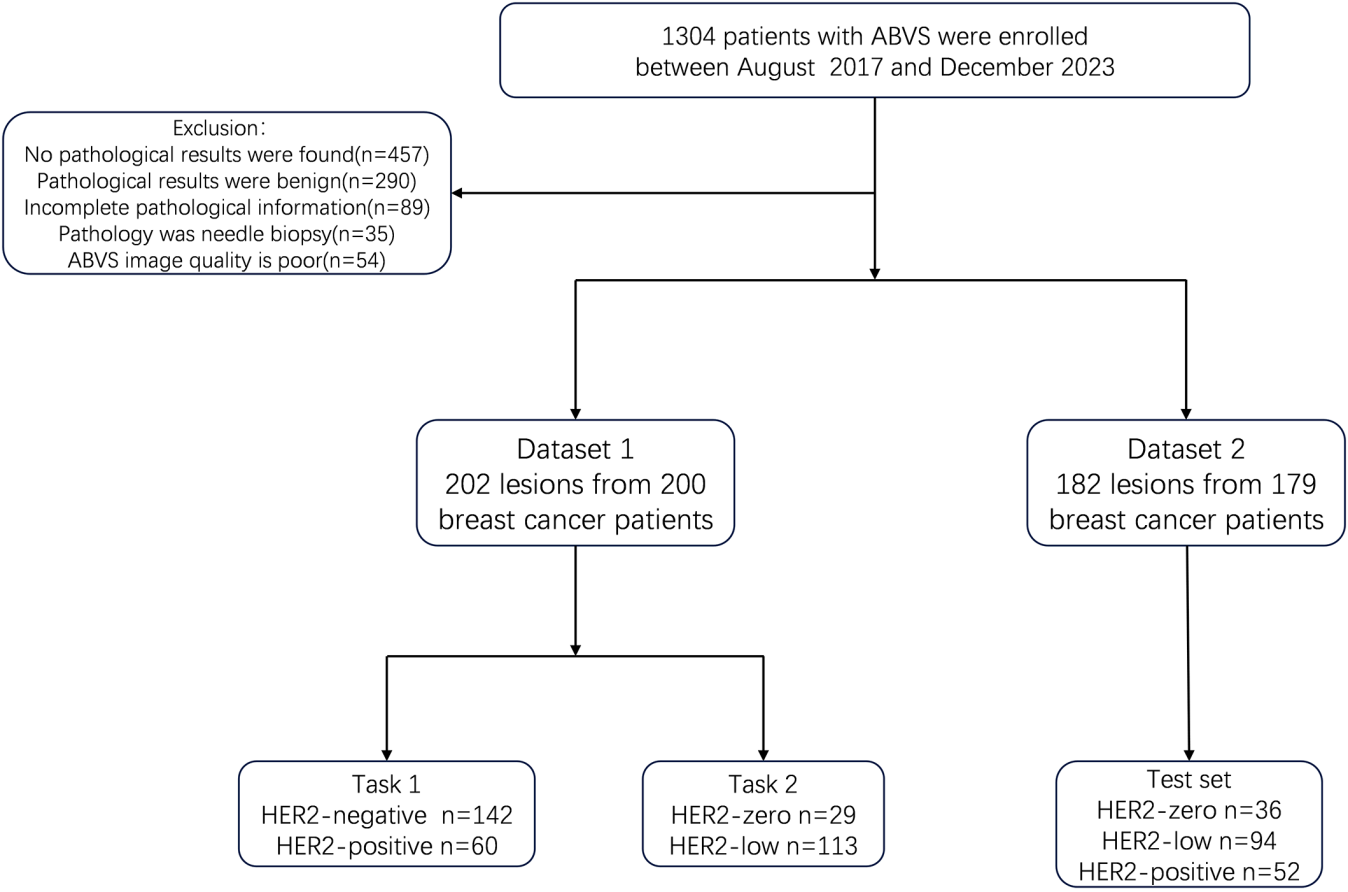
Flowchart of patients and study design. ABVS, Automated Breast Volume scanner; Task 1, the distinction of HER2-positive from HER2-negative breast cancers. Task 2, the distinction of HER2-low from HER2-zero breast cancers.

The in-house data was designated as Dataset 1, while the data from the three external hospitals were categorized as Dataset 2. A total of 384 lesions from 379 patients were collected, including 202 lesions from 200 patients at Hospital 1, 40 lesions from 40 patients at Hospital 2, 24 lesions from 24 patients at Hospital 3, and 118 lesions from 115 patients at Hospital 4. We designed two classification tasks: Task 1 aimed to differentiate HER2-positive from HER2-negative breast cancer, and Task 2 aimed to distinguish HER2-zero from HER2-low expression. Even for multifocal patients, each breast lesion was analyzed independently. In the test set, the three multifocal breast cancer patients each had one lesion per breast, and all six lesions were HER2-low expressing. The patient flowchart is shown in **Figure 1**.

### 2.2 Clinicopathological data

Clinical, ultrasound, and histopathological data were obtained from electronic medical record systems and pathology databases. The dataset included continuous variables: patient age, ultrasound lesion size, pathological lesion size, shear wave velocity (SWV) means, SWV maximum, and SWV minimum. Categorical variables included lymph node (LN) metastasis status on ultrasound, Breast Imaging Reporting and Data System (BIRADS) classification, pathological LN metastasis, and the status of ER, PR, HER2, Ki67, as well as molecular subtypes. Notably, pathological lesion size, SWV mean, SWV maximum, and SWV minimum were only available in Dataset 1, as these variables were not recorded in the other hospitals.

Lesions were classified according to the fifth edition of the American College of Radiology’s BIRADS guidelines. Ultrasound assessment of benign LN requires meeting the following three criteria simultaneously: (1) oval shape with a long-axis/short-axis ratio ≥ 1.5; (2) normal hilum structure, with the cortex presenting as a uniform hypoechoic ring or “C” shape; and (3) sparse and regularly distributed blood flow signals. LN that does not meet these criteria are classified as potentially malignant. The status of ER, PR, and HER2 was determined according to the American Society of Clinical Oncology/College of American Pathologists 2018 guidelines using IHC and FISH. The cutoff value for Ki-67 remains controversial (27, 28). Some studies suggest that a threshold of 20% for Ki-67 more accurately reflects the proliferative status of tumor cells (29). Therefore, this study adopted a Ki-67 threshold of 20%.

### 2.3 ABVS examination and image acquisition

All patients underwent ABVS examinations performed by ultrasound specialists with over five years of experience in breast imaging. The patients were positioned supine with both arms raised, fully exposing the breasts and axillae. A linear array transducer was used to initially locate the lesions, assess and document lesion size, and evaluate LN status. Prior to performing the ABVS scan, the mechanical arm was adjusted to ensure that appropriate pressure was applied by the probe to the breast without causing discomfort. The most suitable scanning mode was selected based on breast size. A 5-14 MHz linear array transducer, approximately 15 cm in width, was then used to apply gentle pressure to the patient’s chest wall, ensuring complete image display. Three volumetric scans were performed from the lower part of the breast to the upper part, covering the anterior, lateral, and medial regions. After the scan, the nipple position was marked, and the collected data were transferred to the ABVS workstation. Three views of the lesion were obtained through 3D reconstruction, allowing simultaneous observation of its morphology in the transverse, sagittal, and coronal planes.

Hospital 4 used the Invenia ABVS system (GE Healthcare, Sunnyvale, CA, USA) for ABVS examinations, while the other three hospitals utilized the Acuson S2000 ultrasound system (Siemens Medical Solutions, Mountain View, CA, USA). Patients in Dataset 1 also underwent Virtual Touch Imaging mode during their examinations to acquire Virtual Touch Imaging data. The breast lesions were scored on a scale from 1 to 5, reflecting the degree of stiffness from soft to hard. Additionally, in Virtual Touch Quantification mode, at least five SWV measurements were taken at the lesion site, and the average SWV value was calculated.

### 2.4 Image segmentation and feature extraction

**Figure 2:**
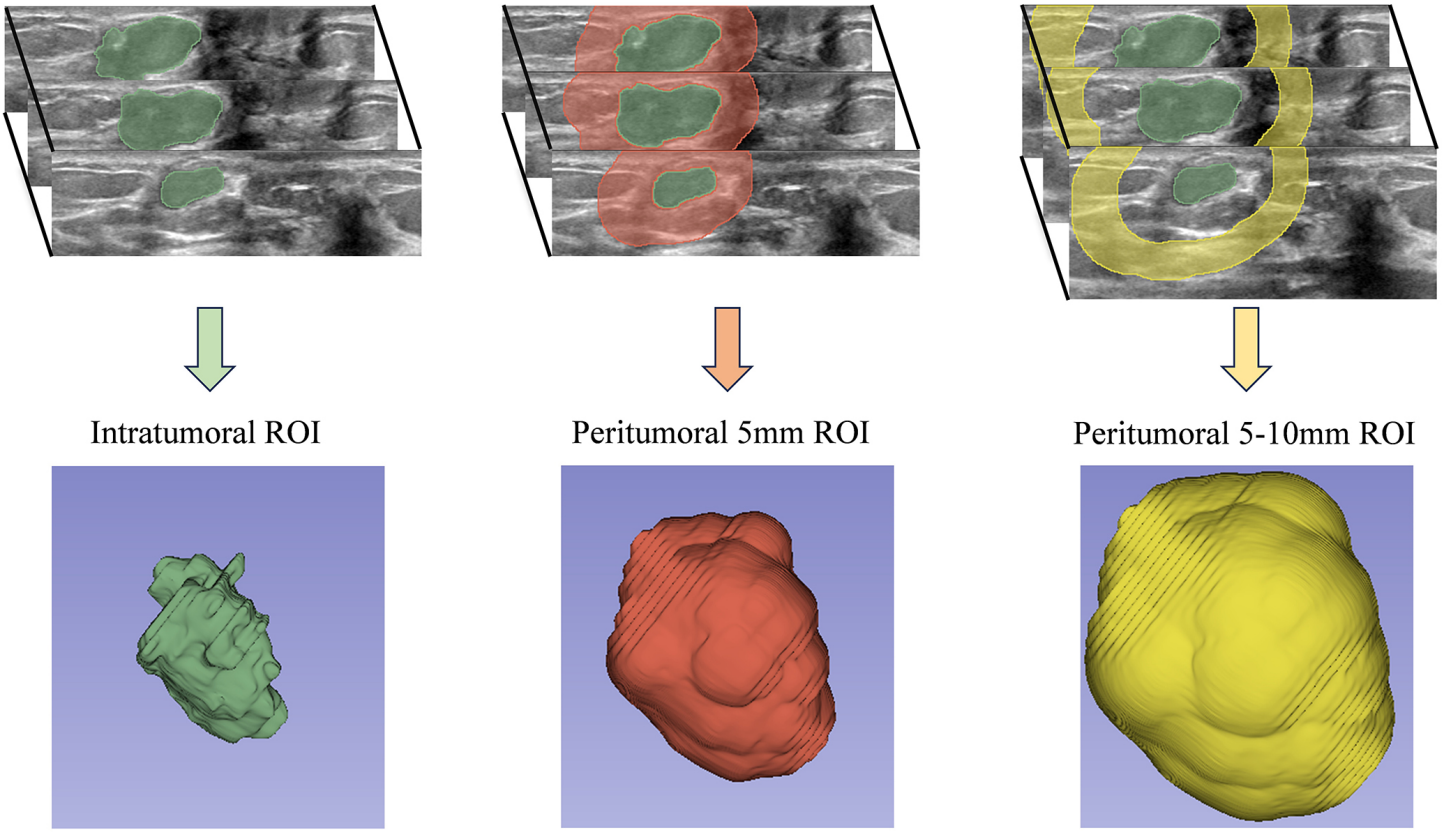
Intratumoral and Peritumoral ROI segmentation workflow.

ABVS images were reviewed by an ultrasound physician with over five years of experience in breast imaging. The ROI was manually segmented, focusing on lesions with corresponding pathological results for patients with multiple breast lesions. The physician was blinded to clinical and pathological data during the segmentation process. In contrast to the majority of prior studies that focused on segmenting the ROI within the largest cross-sectional or longitudinal plane, our research employed the 3D Slicer software (version 5.0.3) for the entire-lesion ROI segmentation of the ABVS volumetric images. our 3D whole-lesion radiomics analysis enables the capture of volumetric heterogeneity that 2D methods may overlook. This is crucial, as breast cancer exhibits spatial variability in texture and morphology, which may correlate with HER2 expression patterns. A total of 1,316 radiomics features were extracted from each ROI, compared to approximately 500 features in 2D ultrasound-based studies (30), demonstrating an enhanced diversity of features. This approach facilitated a more thorough and comprehensive extraction of radiomics features. Using the “Hollow” mode in 3D Slicer, we reconstructed the ROI for the peritumoral region at 5mm and 5-10mm distances around the tumor (**Figure 2**). This method yielded three separate ROI datasets for each lesion: the tumor itself and two peritumoral regions. To evaluate the reproducibility of feature extraction from the ROIs, 50 randomly selected lesions were re-segmented. Intra-observer data were generated by the same physician re-segmenting the same 50 lesions after a two-week interval. Inter-observer data were obtained by a second ultrasound physician, also with over five years of experience in breast imaging, who performed the segmentation while blinded to clinical and pathological outcomes.

Radiomics features related to tumor texture and morphological structure were extracted from the manually delineated ROIs using the Pyradiomics package (version 3.0; https://pypi.org/project/pyradiomics/3.0/) within Python (version 3.7). Prior to feature extraction, to mitigate scanning variability between different hospital ultrasound systems (GE Inventio ABVS and Siemens Accustom S2000), images were resampled to a uniform voxel size of 1 mm³. This step aimed to eliminate the impact of spatial resolution differences caused by varying ABVS slice thickness across institutions on texture features. For intensity normalization, the Z-score method was employed due to its robustness to outliers compared to the Min-Max method, making it more suitable for the gray-scale distribution of medical images (30). Furthermore, features with an Intraclass Correlation Coefficient (ICC) of less than 0.80 were excluded to ensure excellent reproducibility of the remaining features, thereby minimizing variability introduced by segmentation discrepancies. A total of 1,316 radiomics features related to tumor texture and morphology were extracted from each ROI using Python (version 3.7). After excluding features with intra-group and ICC below 0.80, the following features were retained: 1,157 from the tumor ROI, 1,260 from the 5mm peritumoral ROI, and 1,214 from the 5-10-mm peritumoral ROI.

### 2.5 Radiomics feature selection and model construction

For model construction, Dataset 1 was randomly split into a training set and a validation set in a 7:3 ratio. For Task 1, the dataset was divided into a training set (99 HER2-negative and 42 HER2-positive cases), a validation set (43 HER2-negative and 18 HER2-positive cases), and a test set (130 HER2-negative and 52 HER2-positive cases). For Task 2, the dataset comprised a training set (20 HER2-zero and 79 HER2-low cases), a validation set (9 HER2-zero and 34 HER2-low cases), and a test set (36 HER2-zero and 94 HER2-low cases). This stratification ensured a robust evaluation of the models across distinct HER2 expression categories. The comparison between the training and validation sets for two classification tasks is shown in **Supplementary Tables 1** and **2**.

We employed the Synthetic Minority Over-sampling Technique (SMOTE) to address class imbalance in the training set. Specifically, HER2-positive and HER2-zero samples were oversampled exclusively within the training set to generate synthetic samples, thereby balancing the class ratio (adjusted negative-to-positive ratio = 1:1). This approach reduced bias in the feature coefficients. The validation and test sets retained their original data distributions to prevent the introduction of bias from oversampling, ensuring the clinical validity of model evaluation.

During the feature selection process, the correlation between features and the target variable was initially assessed using the t-test. Given the sensitivity of the t-test to data variance, homogeneity of variance was first verified: if the p-value > 0.05, indicating equal variance between groups, a standard independent samples t-test was applied; otherwise, Welch’s t-test was used. Features significantly associated with the target variable (p-value < 0.05) were retained. Subsequently, collinearity analysis was performed: if the data met the normality assumption, Pearson’s correlation coefficient was utilized; otherwise, Spearman’s correlation coefficient was employed. Features with an absolute correlation coefficient > 0.9 relative to other features were excluded to mitigate multicollinearity and prevent model overfitting. The Max-Relevance and Min-Redundancy method was then applied to retain 10 features by maximizing their relevance to the target variable while minimizing redundancy among features, achieving a balance between computational efficiency and model performance. Further feature refinement was conducted using Least absolute shrinkage and selection operator regression, with the regularization parameter (λ) optimized through 10-fold cross-validation to minimize binomial deviation, thereby selecting parsimonious yet predictive non-zero coefficient features and constructing a radiomics score. Lasso feature filtering is shown in **Supplementary Figure 1**. Finally, a logistic regression model with L2 regularization (C = 1.0) was trained based on the radiomics score, and the optimal classification threshold was determined by maximizing Youden’s index. The validation set was used to fine-tune the model parameters for optimal performance. For each classification task, three models were built: Model 1 included radiomics features from the tumor region alone; Model 2 included features from both the tumor region and a 5mm peritumoral region; and Model 3 incorporated features from the tumor region, the 5mm peritumoral region, and the 5-10mm peritumoral region.

The feature selection pipeline employed in this study follows a conventional radiomics workflow (31, 32), which has been widely adopted for effectively screening high-dimensional data. This approach ensures a systematic and rigorous reduction of feature dimensionality while retaining biologically and clinically relevant information.

### 2.6 Model validation

The predictive performance of the test set was used to evaluate the comprehensive performance of the three models constructed for different tasks. We implemented a comprehensive validation strategy to evaluate the performance of the three models across multiple dimensions, ensuring a rigorous assessment of the robustness and generalizability of the radiomics models through various validation steps. Model performance was assessed using the AUC, sensitivity, specificity, and accuracy. The DeLong method was employed to compare AUC values between models and to evaluate statistical significance. Additionally, calibration curves were plotted to assess the agreement between predicted probabilities and observed outcomes.

For clinical utility evaluation, Decision Curve Analysis (DCA) was conducted to quantify the net benefit of using the radiomics model across a range of threshold probabilities, compared to default strategies such as “treat all” or “treat none.” This approach provides a practical assessment of the model’s potential impact on clinical decision-making. Together, these methods ensure a rigorous evaluation of the model’s performance, reliability, and applicability in real-world clinical settings.

### 2.7 Data analysis

Statistical analyses were conducted using R software (version 4.3.2). The ‘CBCgrps’ package was used to assess differences in both categorical and continuous variables between the two groups. The function automatically assessed the distribution of continuous variables, employing independent samples t-tests for normally distributed data, reported as *x*±*s*, and Wilcoxon rank-sum tests for non-normally distributed data, reported as M(IQR). Categorical variables were compared using Fisher’s exact test or Chi-squared tests, presented as n (%). Detailed differences in clinical and pathological characteristics between HER2-negative and HER2-positive patients, as well as between HER2-zero and HER2-low expression patients, were analyzed. ICC was calculated using Python (version 3.7) to assess inter-rater and intra-rater reliability and consistency. Univariate and multivariate logistic regression analyses were conducted using the ‘glm’ package, starting with univariate analysis to identify statistically significant predictive factors, which were then included in the multivariate model. ROC curves were plotted, and DeLong’s test was applied to compare the AUC among the various models, utilizing the ‘pROC’ package. Other metrics, including sensitivity, specificity, and accuracy, were also calculated. Calibration curves were constructed using the ‘rms’ package to evaluate discrepancies between predicted and actual data. The ‘rmda’ package was employed to quantify net benefits at various threshold probabilities, evaluating the clinical value of each model. All statistical tests were two-tailed, with a significance level set at p < 0.05.

## 3 Results

### 3.1 Patient characteristics

According to the inclusion and exclusion criteria, a total of 384 lesions from 379 patients were selected for the study, with an overall average age of 56.03 ± 11.22 years. The average lesion diameter was 2.2(1.6,2.8) cm. Among these patients, 112 exhibited high HER2 expression (29.2%), 207 had HER2-low expression (53.9%), and 65 showed HER2-zero expression (16.9%). In terms of molecular subtypes, 288 cases (75%) were classified as luminal types, including 78 cases of luminal A (20%), 68 cases of luminal B-HER2-positive (18%), and 142 cases of luminal B-HER2-negative (37%). Additionally, 44 cases (11%) were HER2-positive, and 52 cases (14%) were triple-negative breast cancer.

Preoperative ultrasound differences between Datasets 1 and 2 were observed in ultrasound mass size and BIRADS scores (**Table 1**). These differences can be attributed to the higher number of cases from Cancer Hospital in Dataset 2, where patients presented with a greater degree of malignancy, larger lesion diameters, and more ultrasound-detected metastatic LN, leading to higher BIRADS scores.

**Table 1:** Analysis of clinical factors differences between dataset 1 and dataset 2 patients.

Variables	Total (n=384)	Dataset 1 (n=202)	Dataset 2 (n=182)	P
Age (years), Mean ± SD	55.38 ± 11.15	56.42 ± 10.85	54.21 ± 11.39	0.053
US size (cm), Median (Q1,Q3)	2.2 (1.6,2.8)	2.1 (1.5,2.6)	2.3 (1.7,2.9)	**0.016**
USLN,n (%)				0.058
Negative	272 (71)	152 (75)	120 (66)	
Positive	112 (29)	50 (25)	62 (34)	
PatLN,n (%)				1
Negative	239 (62)	126 (62)	113 (62)	
Positive	145 (38)	76 (38)	69 (38)	
BIRADS,n (%)				0.037
3	1 (0)	0 (0)	1 (1)	
4A	34 (9)	18 (9)	16 (9)	
4B	99 (26)	49 (24)	50 (27)	
4C	162 (42)	98 (49)	64 (35)	
5	88 (23)	37 (18)	51 (28)	
Convergence,n (%)				0.637
Negative	222 (58)	114 (56)	108 (59)	
Positive	162 (42)	88 (44)	74 (41)	
ER,n (%)				0.045
Negative	101 (26)	44 (22)	57 (31)	
Positive	283 (74)	158 (78)	125 (69)	
PR,n (%)				0.379
Negative	140 (36)	69 (34)	71 (39)	
Positive	244 (64)	133 (66)	111 (61)	
HER2,n (%)				0.362
Zero	65 (17)	29 (14)	36 (20)	
Low	207 (54)	113 (56)	94 (52)	
Positive	112 (29)	60 (30)	52 (29)	
KI67 (20),n (%)				<0.001
Low	105 (27)	33 (16)	72 (40)	
High	279 (73)	169 (84)	110 (60)	
Molecular subtype,n (%)				
HER-2+	44 (11)	16 (8)	28 (15)	
LA	78 (20)	24 (12)	54 (30)	
LB-	142 (37)	92 (46)	50 (27)	
LB+	68 (18)	44 (22)	24 (13)	
TN	52 (14)	26 (13)	26 (14)	

SD, standard deviation; USsize, ultrasound image lesion size; USLN, ultrasound-reported lymph node status; PatLN, pathologically confirmed lymph node metastasis; BIRADS, Breast Imaging Reporting and Data System; ER, estrogen receptor; PR, progesterone receptor; HER2, human epidermal growth factor receptor-2.

Bold represents statistically significant difference.

### 3.2 Construction of radiomics models

For the classification task distinguishing between HER2-negative and HER2-positive expression, the final Model 1 selected 4 features from the tumor region. Model 2 included a total of 10 features, comprising 4 from the tumor region and 6 from the 5mm peri-tumoral region. Model 3 also comprised 10 features, with 2 from the tumor region, 4 from the 5mm peri-tumoral region, and 4 from the 5-10mm peri-tumoral region.

For the classification task differentiating between HER2-zero and low expression, the final Model 1 selected 5 features from the tumor region. Model 2 included 8 features, consisting of 3 from the tumor region and 5 from the 5mm peri-tumoral region. Model 3 selected a total of 10 features, comprising 1 from the tumor region, 5 from the 5mm peri-tumoral region, and 4 from the 5-10mm peri-tumoral region

For classification Task 1, the model that combines tumor and 5-mm peri-tumoral regions also performed the best. In the radiomics score formula, features are prefixed with “0” and “5,” where “0” denotes the tumor region and “5” represents the 5 mm peritumoral region. The radiomics model was calculated using the following formula:


Rad score=−1.10728445

$$+0\_\text{wavelet}-\text{H}\text{L}\text{L}\_\text{g}\text{l}\text{c}\text{m}\_\text{Correlation}*\left(-0.02471191\right)$$

+0_wavelet−LHH_glcm_Imc2∗0.20903593

$$+5\_\text{square}\_\text{glrlm}\_\text{ShortRunLowGrayLevelEmphasis}*\left(-0.01451517\right)$$

+5_wavelet−HHH_gldm_DependenceEntropy∗0.09835993

$$+5\_\text{wavelet}-\text{LLL}\_\text{firstorder}\_\text{Skewness}*\left(-0.20506020\right)$$

+5_wavelet−HLL_glcm_Imc2∗0.11135310

$$+5\_\text{wavelet}-\text{HLL}\_\text{firstorder}\_\text{Kurtosis}*\left(-0.20027030\right)$$

+0_original_shape_Elongation∗0.23623459

$$\begin{array}{c} +0\_\text{wavelet}-\text{HLL}\_\text{glszm}\_\text{SmallAreaHighGrayLevelEmphasis}\\ {}*0.30223322 \end{array}$$

+5_wavelet−HLL_firstorder_Skewness∗0.26365304

For classification Task 2, the model that combines tumor and 5-mm peri-tumoral regions also performed the best. In the radiomics score formula, features are prefixed with “0” and “5,” where “0” denotes the tumor region and “5” represents the 5 mm peritumoral region. The radiomics model was calculated using the following formula:


Rad score=1.609161316

+5_wavelet−HLH_firstorder_Skewness∗0.009526300

$$\begin{array}{c} +5\_\text{wavelet}-\text{HHH}\_\text{glrlm}\_\text{RunLengthNonUniformityNormalized}\\ {}*0.014674600 \end{array}$$

$$+5\_\text{wavelet}-\text{HLH}\_\text{glcm}\_\text{InverseVariance}*\left(-0.034904310\right)$$

+0_wavelet−LLH_glcm_Correlation∗0.051930824

$$+0\_\text{squareroot}\_\text{firstorder}\_\text{Skewness}*\left(-0.006017558\right)$$

$$\begin{array}{c} +5\_\text{wavelet}-\text{HHH}\_\text{glszm}\_\text{LowGrayLevelZoneEmphasis}\\ {}*0.110547153 \end{array}$$

+0_wavelet−HHL_firstorder_Skewness∗0.212455284

$$\begin{array}{c} +5\_\text{wavelet}-\text{HLH}\_\text{glszm}\_\text{SmallAreaHighGrayLevelEmphasis}\\ {}*0.189145205 \end{array}$$

### 3.3 Model validation

**Figure 3:**
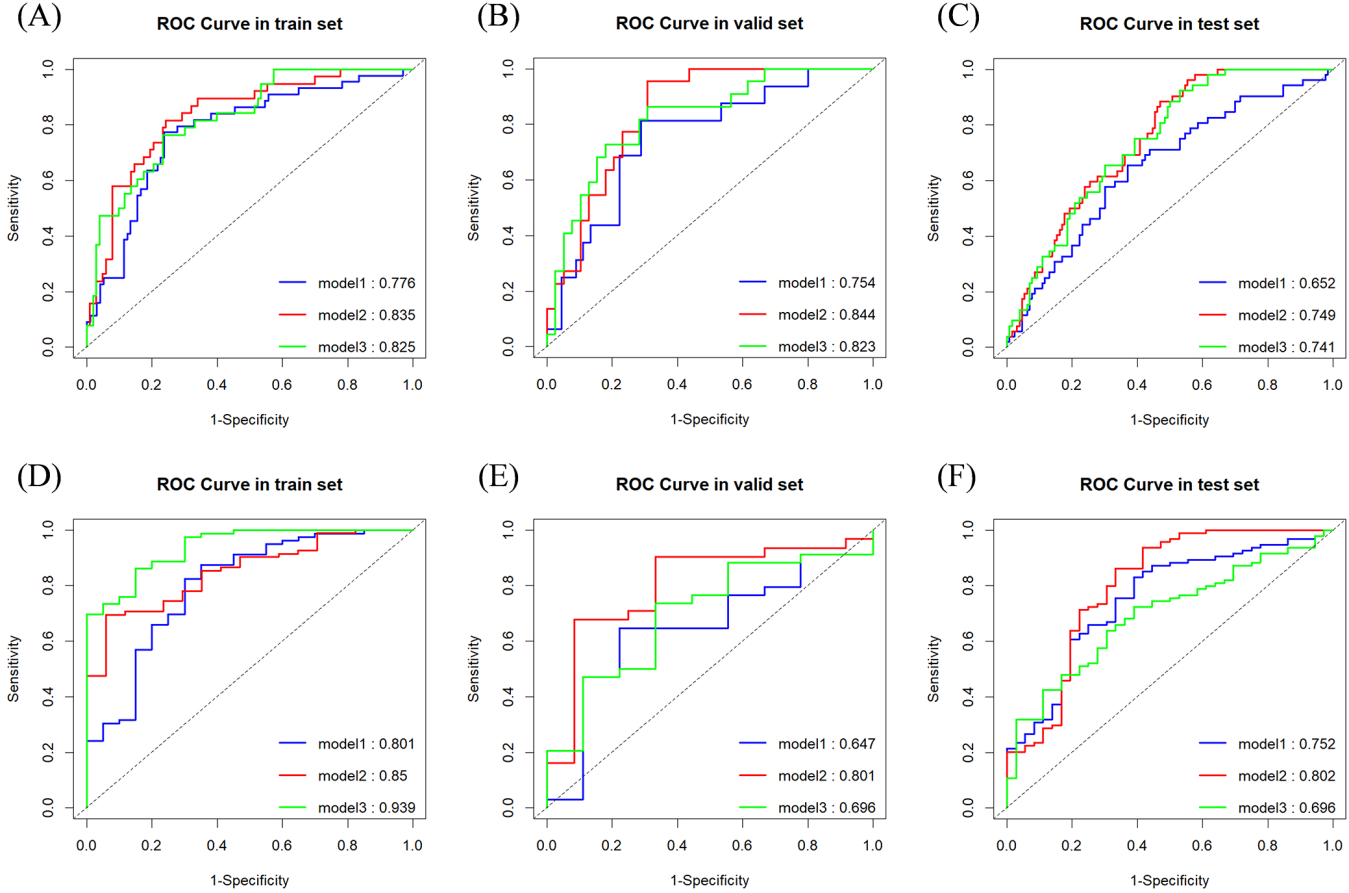
ROC curves and area under the curve for classification tasks. **(A)** Task 1 (Training Set); **(B)** Task 1 (Validation Set); **(C)** Task 1 (Test Set); **(D)** Task 2 (Training Set); **(E)** Task 2 (Validation Set); **(F)** Task 2 (Test Set).

**Table 2** summarizes the performance metrics of the models developed for the two classification tasks, while the ROC curves for the three models in both tasks are depicted in **Figure 3**.

**Table 2:** Comparison of radiomics models for two classification tasks.

Radiomics Signatures	Cohorts	AUC	ACC	SPE	SEN	P_2	P_3
Task 1							
model1	Training	0.777	0.766	0.763	0.773	0.302	0.395
	Validation	0.754	0.738	0.711	0.813	0.297	0.445
	Test	0.652	0.637	0.631	0.654	**0.016**	0.060
model2	Training	0.836	0.773	0.757	0.816		0.633
	Validation	0.844	0.787	0.692	0.955		0.564
	Test	0.749	0.632	0.531	0.885		0.742
model3	Training	0.825	0.766	0.767	0.763		
	Validation	0.823	0.754	0.692	0.864		
	Test	0.741	0.599	0.469	0.923		
Task 2							
model1	Training	0.801	0.828	0.650	0.873	0.513	**0.021**
	Validation	0.647	0.674	0.778	0.647	0.248	0.605
	Test	0.752	0.769	0.611	0.830	0.250	0.192
model2	Training	0.850	0.737	0.941	0.695		0.081
	Validation	0.801	0.744	0.917	0.677		0.408
	Test	0.802	0.808	0.667	0.862		**0.038**
model3	Training	0.939	0.859	0.850	0.861		
	Validation	0.696	0.721	0.667	0.735		
	Test	0.696	0.692	0.611	0.723		

Task 1, HER2-negative and HER2-positive classification; Task 2, HER2-zero and HER2- low classification.

Model 1 included radiomics features from the tumor region alone; Model 2 included features from both the tumor region and a 5mm peritumoral region; Model 3 incorporated features from the tumor, 5mm peritumoral region, and 5-10mm peritumoral region.

AUC, area under the receiver operating characteristic curve; ACC, accuracy; SPE, specificity; SEN, sensitivity.

P_2: DeLong Test with Model 2; P_3: DeLong Test with Model 3.

Bold represents statistically significant difference.

For classification task 1, Model 2 demonstrated superior predictive performance across multiple datasets. In the validation set, it achieved the highest AUC (0.844) (**Figure 3B**), exceptional sensitivity (0.955), and satisfactory accuracy (0.787), underscoring its robustness in identifying true positive cases. Notably, in the test set, it outperformed the other models with an AUC of 0.749 (**Figure 3C**) and a sensitivity of 0.885, highlighting its potential for reliable clinical application.

For classification task 2, Model 2 exhibited superior performance across multiple datasets, particularly in the test set, where it achieved the highest AUC (0.802) (**Figure 3F**), sensitivity (0.862), and accuracy (0.808) compared to the other models. Additionally, (**Figures 3D, E**) showed consistent performance in both the training set (AUC 0.850) and validation set (AUC 0.801), indicating its generalizability. Model 2 successfully predicted all six lesions from the three multifocal breast cancer patients in the test set as HER2-low expressing. These findings underscore the added value of incorporating peritumoral radiomics features within a 5mm margin, which enhances predictive performance over the intratumoral only model.

In Task 1, although no statistically significant differences were observed between Models 1 and 3 or between Models 2 and 3 in the test set, a notable distinction was identified between Models 1 and 2. This finding suggests that Model 2 outperformed both Models 1 and 3.

In Task 2, the DeLong test showed no significant differences between Models 1 and 2 or between Models 2 and 3 in the training set. However, a significant difference was observed between Models 1 and 3, suggesting that Model 3, which extracted a greater number of features from both the tumor and its surrounding tissues, demonstrated superior performance during training. Conversely, in the test set, a significant difference was found between Models 2 and 3, with Model 3 performing markedly worse than Model 2. These findings indicate that Model 3 may have overfitted to the training data. It is suspected that the features selected for Model 3 were highly correlated with the training set, with a substantial proportion derived from the peritumoral regions. Including an extensive peritumoral area may introduce extraneous tissue, negatively affecting performance during external validation, particularly when images are acquired from different imaging systems.

### 3.4 Calibration and decision curves for two classification tasks

**Figure 4:**
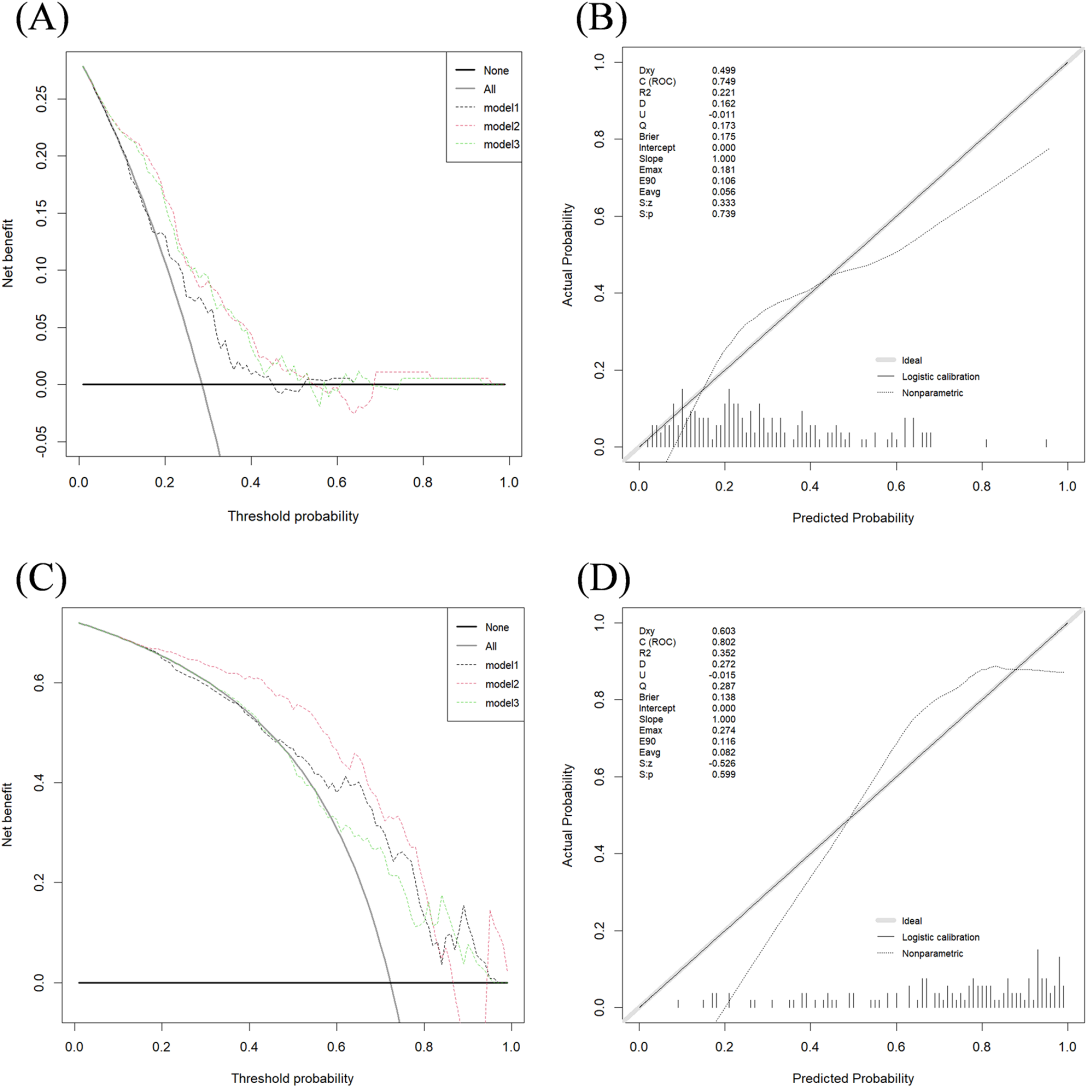
DCA performance of three models in two classification tasks and calibration curve of model 2. **(A)** Decision curve for Task 1 test set; **(B)** Calibration curve for Model 2 in Task 1 test set; **(C)** Decision curve for Task 2 test set; **(D)** Calibration curve for Model 2 in Task 2 test set. The x-axis and y-axis show the threshold probability and the net benefit, respectively. The light black line shows the assumption that all breast cancer patients were HER2-negative or HER2-zero (the strategy of “treat all”). The bold black line shows the assumption that all breast cancer patients were HER2-negative or HER2-zero (the strategy of “treat none”).

**Figure 4** presents the calibration curves and DCA for the two classification tasks using Dataset 2. The calibration curves and DCA of all models are shown in **Supplementary Figures 2–4**.

The calibration curves indicate strong agreement between the predicted and actual classification results across the training, testing, and validation sets for both tasks.

The DCA curve for Task 1 (**Figures 4A, B**) shows that Model 1 provides a net benefit across a wide probability threshold range (0.19–0.45; 0.53–0.63), Model 2 offers a net benefit across a broader probability range (0.05–0.54; 0.69–0.94), and Model 3 demonstrates a net benefit across a wide threshold range (0.05–0.53; 0.76–0.93). All three models deliver higher net benefits than the “treat none” or “treat all” strategies.

For Task 2 (**Figures 4C, D**), the DCA curve shows that Model 1 provides a net benefit across a wide threshold probability range (0.49–0.94), Model 2 offers a net benefit within a broader probability range (0.11–0.89), and Model 3 shows a net benefit across two distinct probability ranges (0.32–0.40; 0.63–0.94). Similar to Task 1, all models outperformed the “treat none” and “treat all” strategies, providing valuable insights for clinical decision-making.

In both Task 1 and Task 2, the DCA graphs show that Model 2 exhibits a wider range of positive net benefit values compared to Models 1 and 3. This suggests that Model 2 is better equipped to account for a broader spectrum of clinical scenarios, ultimately enabling patients to derive maximum benefit from the predictive modeling.

### 3.5 Univariate and multivariate analysis of clinical and ultrasound variables

**Table 3** presents a comparative analysis of the clinical and pathological characteristics between patients with different HER2 statuses in Dataset 1. We aim to identify independent predictive factors for HER2 expression levels through the analysis of clinical and ultrasound variables, and subsequently integrate these factors with the radiomics model. This combined approach seeks to enhance the predictive accuracy and clinical utility of the model, providing a more comprehensive tool for assessing HER2 status in breast cancer.

**Table 3:** Univariate analysis of clinical factors distinguishing HER2 status in dataset 1.

Variables	HER2-Negative VS. Positive		P	HER2-Zero VS. Low		P
	HER2-Negative (n=142)	HER2-Positive (n=60)		HER2-Zero (n=29)	HER2-Low (n=113)	
Age (years),Mean ± SD	56.69 ± 11.36	55.78 ± 9.61	0.563	56.62 ± 14.73	56.71 ± 10.4	0.976
USsize (cm),Median (Q1,Q3)	2 (1.4,2.58)	2.35 (1.7,2.8)	**0.008**	2.1 (1.4,2.7)	2 (1.5,2.5)	0.548
Patsize (cm),Median (Q1,Q3)	2 (1.42,2.6)	2.5 (2,3)	**0.002**	2 (1.3,2.7)	2 (1.5,2.6)	0.911
USLN,n (%)			0.097			0.407
Negative	112 (79)	40 (67)		25 (86)	87 (77)	
Positive	30 (21)	20 (33)		4 (14)	26 (23)	
PatLN,n (%)			1			0.471
Negative	89 (63)	37 (62)		16 (55)	73 (65)	
Positive	53 (37)	23 (38)		13 (45)	40 (35)	
BIRADS,n (%)			0.318			0.605
4A	13 (9)	5 (8)		2 (7)	11 (10)	
4B	37 (26)	12 (20)		10 (34)	27 (24)	
4C	63 (44)	35 (58)		13 (45)	50 (44)	
5	29 (20)	8 (13)		4 (14)	25 (22)	
SE,n (%)			0.65			0.435
2	3 (2)	1 (2)		0 (0)	3 (3)	
3	19 (13)	8 (13)		2 (7)	17 (15)	
4	31 (22)	18 (30)		5 (17)	26 (23)	
5	89 (63)	33 (55)		22 (76)	67 (59)	
SWVmean (m/s),Mean ± SD	5.07 ± 1.67	5.21 ± 1.63	0.578	5.27 ± 1.74	5.02 ± 1.65	0.489
SWVmax (m/s),Median (Q1,Q3)	6.33 (4.86,8.27)	6.28 (4.86,8.01)	0.957	7.14 (5.24,8.65)	6.31 (4.72,8.26)	0.387
SWVmin (m/s),Median (Q1,Q3)	3.82 (3.03,4.59)	4.03 (3.11,4.77)	0.52	3.97 ± 1.18	3.85 ± 1.25	0.64
Convergence,n (%)			0.039			0.28
Negative	73 (51)	41 (68)		18 (62)	55 (49)	
Positive	69 (49)	19 (32)		11 (38)	58 (51)	
ER,n (%)			0.201			0.008
Negative	27 (19)	17 (28)		11 (38)	16 (14)	
Positive	115 (81)	43 (72)		18 (62)	97 (86)	
PR,n (%)			<0.001			0.105
Negative	35 (25)	34 (57)		11 (38)	24 (21)	
Positive	107 (75)	26 (43)		18 (62)	89 (79)	
KI67 (20),n (%)			0.027			0.211
Low	29 (20)	4 (7)		3 (10)	26 (23)	
High	113 (80)	56 (93)		26 (90)	87 (77)	
Molecular subtype,n (%)						
HER2+	0 (0)	16 (27)		3 (10)	21 (19)	
LA	24 (17)	0 (0)		16 (55)	76 (67)	
LB-	92 (65)	0 (0)		10 (34)	16 (14)	
LB+	0 (0)	44 (73)		(n=29)	(n=113)	
TN	26 (18)	0 (0)		56.62 ± 14.73	56.71 ± 10.4	

SD, standard deviation; USsize, ultrasound image lesion size; Patsize, lesion size of pathological specimens; USLN, ultrasound-reported lymph node status; PatLN, pathologically confirmed lymph node metastasis; BIRADS, Breast Imaging Reporting and Data System; SE, strain elastography score; SWV, shear wave elastography; ER, estrogen receptor; PR, progesterone receptor; HER2, human epidermal growth factor receptor-2

Bold represents statistically significant difference.

The analysis comparing HER2-negative and HER2-positive breast cancer patients revealed statistically significant differences in several parameters, including tumor diameter, convergence sign, PR status, and Ki-67 expression status (p < 0.05). For preoperative predictions, ultrasound assessment of tumor diameter and convergence sign under ABVS showed significant relevance. However, no significant differences were found for age, LN metastasis, BIRADS score, elasticity score, SWV mean, SWV maximum, SWV minimum, and ER status (p > 0.05).

The analysis of HER2-low expression breast cancer patients and those with HER2-zero revealed no statistically significant differences in the following variables: age, lesion diameter, LN metastasis, BIRADS score, elasticity score, SWV minimum, SWV mean, SWV maximum, presence of convergence sign, PR status, and Ki-67 status (p > 0.05). A significant difference was found only in ER status (p < 0.05); however, it cannot be used for preoperative prediction.

**Table 4** presents a multivariate analysis of key preoperative factors, including ultrasound-assessed tumor diameter, convergence sign, and radiomics scores associated with clinical pathological characteristics. The findings show that, among these variables, only the radiomics score demonstrates a statistically significant ability to differentiate between HER2-negative and HER2-positive expression. We believe that the radiomics features effectively capture information such as ultrasound tumor size and the convergence sign around the tumor.

**Table 4:** Multivariate logistic regression analysis for distinguishing HER2-negative and HER2-psositive expression in Dataset 1.

Variables	B	SE	Wald	OR(CI)	P
Convergence	-0.646	0.391	2.729	0.524(0.239~1.116)	0.0986
USsize	0.221	0.159	1.915	1.247(0.919~1.733)	0.1664
Radscore	2.327	0.4	33.909	10.25(4.964~23.956)	**<0.001**

OR, odds ratio; CI, confidence interval; USsize, ultrasound image lesion size.

Bold represents statistically significant difference.

## 4 Discussion

An increasing body of research shows that radiomics models constructed using both intratumoral and peritumoral features outperform those relying solely on intratumoral characteristics. Moreover, the optimal peritumoral region varies depending on the cancer location and the specific predictive objectives (33–35). A study aimed at predicting Ki-67 expression levels in breast cancer demonstrated that combining intratumoral features with a 10mm peritumoral region provided the best predictive capability (19). Additionally, radiomics based on Contrast-Enhanced Spectral Mammography, incorporating the intratumoral region plus a 5mm peritumoral area, has shown promising results, achieving a maximum AUC of 0.85 for predicting neoadjuvant chemotherapy response in breast cancer (36).

This study focused on improving the non-invasive preoperative prediction of HER2 expression levels by extracting radiomics features from the tumor region as well as the 5mm and 10mm peritumoral zones to construct various radiomics models. Ultimately, our findings revealed that the integration of intratumoral regions with 5mm peritumoral areas provided the highest performance for both classification tasks. This moderate extension into the peritumoral space facilitates the acquisition of relevant microenvironmental data, including factors such as peritumoral edema (37), tumor-stroma interactions (38), and immune cell infiltration. Furthermore, peritumoral radiomics features can provide insights into the biological characteristics and pathological responses of HER2-positive breast cancer to preoperative targeted therapy by detecting biological information related to lymphocytic spatial structure, vascular invasion, and the immune response of surrounding breast tissue (39). This study also confirms that peritumoral characteristics are not only capable of distinguishing HER2-negative from HER2-positive expressions but also of effectively differentiating between HER2-zero and HER2-low expression cases.

In both classification tasks, a common issue observed was a slight decrease in AUC for the test set compared to the training set. We hypothesize that this decline may be due to the fact that the majority of the test set images were obtained using the Invenia ABVS ultrasound system at Hospital 4, which produces imaging results that differ slightly from those of the Acuson S2000 ultrasound systems used in the other hospitals. Additionally, the relatively small training sample size could also be a contributing factor. However, the comparable AUC levels between the training and test sets suggest that the tumor and peritumoral combination model developed in this study maintains good generalizability, even in the presence of imaging discrepancies. This conclusion is further supported by the decision curves and calibration plots, which also demonstrate the model’s robustness.

In radiomics research, SMOTE has been widely utilized to handle small sample sizes and imbalanced data (30). Compared to simple oversampling methods, such as random duplication, SMOTE generates new samples through interpolation, better preserving the biological significance of tumor heterogeneity features, such as texture and morphology. Although this algorithm was employed in the model development, we observed a phenomenon of high sensitivity and low specificity in the test set in this study, which is also commonly seen in the evaluation metrics of other models. We provide the following analysis for this observation: First, the imbalance in the training set data, which was addressed through oversampling, may have prioritized overall model performance at the expense of specificity. Second, Due to the limited sample size of the overall dataset, the training set was relatively small and exclusively comprised images from a single ultrasound device. whereas approximately two-thirds of the test set cases were acquired using a different ultrasound device. This limited the model’s exposure to diverse data sources, contributing to the observed lower specificity.

In breast cancer treatment, HER2-positive patients require targeted therapies (e.g., trastuzumab), making the cost of missed diagnoses (low sensitivity) far greater than that of false positives (low specificity). Therefore, high sensitivity (88.5%) holds greater clinical value. The disparity in imaging devices between the training and test sets underscores the importance of incorporating heterogeneous data sources to enhance model generalizability and performance. In future studies, we aim to collect more data from this imaging device to incorporate into the training set, which may resolve the current issues and further improve model performance.

The integrated model proposed in this study shows promise as a non-invasive approach for facilitating personalized clinical diagnosis and treatment strategies. In the HER2-negative versus HER2-positive classification task, the test set AUC reached 0.749, surpassing the performance of an earlier radiomics model developed using dynamic contrast-enhanced MRI, which reported an AUC of 0.713 (35). For the HER2-low expression classification task, our model exhibited moderate predictive capability, achieving an AUC of 0.850 in the training dataset, 0.802 in the validation dataset, and 0.801 in the testing dataset. In contrast, a previous study using a radiomics model based on T1-weighted imaging and apparent diffusion coefficient sequences achieved AUC values of 0.820 in the training dataset, 0.776 in the validation dataset, and 0.711 in the testing dataset (40). Our model’s predictive performance not only surpasses that of earlier studies but also benefits from a larger test set (130 cases in our study versus 43 cases in the previous study). Additionally, this study integrated imaging data from different equipment across multiple hospitals, which provides more robust and convincing results.

The prediction results for the three multifocal breast cancer patients in the test set were analyzed. All three patients had bilateral breast lesions, and all six lesions were identified as HER2-low expressing. Model 2 successfully predicted these six lesions correctly, which holds significant clinical implications for treatment guidance. Although literature suggests that approximately 5-10% of multifocal breast cancer patients exhibit HER2 heterogeneity across different lesions (41–43), the multifocal cases in this study were limited and demonstrated consistent HER2 expression. Further in-depth analysis of HER2 expression heterogeneity in multifocal patients will require an expanded cohort of multifocal cases. Around 50% of breast cancer patients classified as HER2-negative display low HER2 expression, thereby restricting their available treatment options. A common characteristic of biomarkers is that their assessment primarily guides treatment decisions rather than defining new biological subtypes. Research has confirmed substantial differences between HER2-low and HER2-zero breast cancers regarding Ki-67 expression levels, hormone receptor status, and mutation rates in the PI3K-Akt signaling pathway (8), disease-free survival, overall survival (44), and response to neoadjuvant chemotherapy (7). Novel ADCs have provided novel targeted treatment strategies for patients with HER2-low breast cancer (45–47). T-DXd has demonstrated manageable safety (46) and superior efficacy in terms of tumor shrinkage and prolonged survival in HER2-low expressing breast cancers (47), providing significant therapeutic benefits. Defining the HER2-low expressing subgroup of breast tumors enables both triple-negative breast cancers and hormone receptor-positive to benefit from T-DXd, signaling potential major advances in future treatment algorithms.

In current clinical practice, invasive procedures such as core needle biopsy are essential for diagnosing breast cancer and determining HER2 status through IHC. Due to the intrinsic heterogeneity of HER2 expression, the diagnosis of HER2-low breast cancer based on core needle biopsy results is prone to errors. Approximately 15.3% of cases are incorrectly categorized as HER2-negative, while 7.3% are misclassified as HER2-positive (48). A recent study revealed that in patients with HER2-negative primary breast cancer, residual disease was observed following neoadjuvant chemotherapy, the overall rate of HER2 status change was 32.4%, including 1.1% shifting from HER2-negative to HER2-positive, transition rates included 15.4% shifting from HER2-low to HER2-zero and 15.9% moving from HER2-zero to HER2-low (49). These findings underscore the importance of closely monitoring HER2 expression changes during the management of HER2-negative breast cancer. Besides providing a non-invasive method for predicting preoperative HER2 expression levels, our model can assist in improving the accuracy of HER2 assessment in core needle biopsy specimens. This enhances the reliability of biopsy pathology results, providing more precise guidance for clinical treatment decisions tailored to each patient’s specific condition, thereby ensuring appropriate therapeutic interventions.

The excellent reproducibility of ABVS minimizes dependence on the clinical expertise of radiologists for procedures such as scanning, equipment adjustments, and image archiving, thereby improving both the standardization and precision of examinations (50). Previous research has shown that ABVS is comparable to MRI in its effectiveness for measuring tumor size (17, 51). These studies have shown that ABVS offers distinct advantages for observing breast lesions, providing strong justification for the implementation of 3D target delineation in the present study.

In this study, when predicting HER2-negative and HER2-positive status, tumor size and the retraction phenomenon assessed by ABVS were significant in both univariate and multivariate clinical-pathological analyses in the training set (p < 0.05). The retraction phenomenon, a typical feature of ABVS in the coronal plane, is particularly effective in visualizing the infiltrative growth of breast cancer. Previous research has shown that ABVS significantly enhances the accuracy of early detection and complicates the diagnosis of breast cancer in dense breast tissue due to the occurrence of this retraction phenomenon (52). The growth of fibrous tissue surrounding the breast tumor, combined with tumor tissue infiltration and traction, leading to the surrounding tissue being drawn toward the tumor, resulting in a radial high-echo band surrounding the tumor on ABVS images. The retraction phenomenon can limit the rapid invasion and distant metastasis of breast cancer cells, allowing the immune system more time to respond to the tumor (53). Tumor size on ABVS has been shown to be significantly linked to ALNM in patients with clinical T1-T2 breast cancer (54). This study also revealed the importance of these two indicators in predicting HER2 levels in breast cancer. However, when these two risk factors were incorporated into the multivariate logistic regression analysis alongside the developed radiomics score, their p-values were > 0.05. This phenomenon has been observed in other studies, such as in predicting the benign or malignant nature of breast tumors and predicting whether breast cancer has ALNM (21, 55). We believe that the radiomics data extracted from the raw ultrasound images can fully capture the tumor and peritumoral information.

In this study, the radiomics features primarily derived from texture characteristics, such as the Gray-Level Co-occurrence Matrix and first-order statistical metrics, with most of the texture features obtained through wavelet transformations. Wavelet-based texture features have been shown in previous studies to hold significant value in the diagnosis of tumor lesions (56). The key benefit of wavelet transformation in image analysis lies in its ability to perform multi-scale analysis, enabling the extraction of texture details across different levels of granularity. Additionally, it demonstrates sensitivity to direction, which facilitates the precise identification of texture changes across various directions. Moreover, its time-frequency localization characteristics enable the accurate detection of localized variations within the image. Furthermore, wavelet transformation improves image contrast, demonstrates a notable degree of noise resistance, and efficiently compresses image information, thereby enhancing the robustness and effectiveness of feature extraction (57). Through the quantification of texture variations in breast lesions, we effectively identified subtle heterogeneity within the lesions, enabling effective differentiation of HER2 expression levels in the tumors.

This study has several strengths. It includes a multi-center external validation cohort with data from multiple hospitals using different ABVS machines and settings, demonstrating the generalizability of the radiomics models developed and confirming their clinical applicability. Moreover, unlike the traditional 2D segmentation of ultrasound images, we conducted segmentation of ABVS images and obtained three-dimensional lesion features for analysis, providing a more comprehensive representation of the tumor’s characteristics. Additionally, the consistency of the extracted features was assessed using the ICC before feature selection, further enhancing the reliability of the radiomics models.

This study has several limitations: (1) Being a retrospective study, it is inherently prone to potential selection bias that may be unavoidable. (2) Although patients were recruited from four centers, the sample size remains relatively small, particularly for subgroups such as multifocal cases or HER2-low expressing lesions. This limitation may affect the generalizability of our findings. While the current model demonstrated acceptable predictive performance (AUC: 0.76–0.79), we acknowledge that the lack of harmonization algorithms, such as ComBat, may introduce variability. This is particularly relevant given that two-thirds of the test set images were acquired using a different scanner than the training set. In subsequent studies, we plan to expand the dataset to include more cases from diverse imaging devices, integrate ComBat harmonization to reduce inter-scanner variability, and incorporate multi-scanner data into the training set to improve model generalizability. (3) Currently, the delineation of target regions is performed manually, which can be time-consuming for three-dimensional images. Future efforts will consider adopting semi-automated or fully automated contouring methods to minimize the complexity of model application and improve prediction speed. (4) Although significant differences in clinical and ultrasound features were observed in univariate analyses, a high correlation and collinearity with omics scores were noted. Future analyses will incorporate additional clinical pathological factors, such as serological markers, to refine the model further.

## Data Availability

The raw data supporting the conclusions of this article will be made available by the authors, without undue reservation.
