# Supplementary material for: Intratumoral and peritumoral radiomics based on automated breast volume scanner for predicting human epidermal growth factor receptor 2 status
Source: Front Oncol. 2025 Apr 16;15:1556317. doi: 10.3389/fonc.2025.1556317 (PMC12041018; doi:10.3389/fonc.2025.1556317)
Supplement: Supplementary file 1 [file DataSheet1.docx]

Supplementary Material

# Supplementary Figures and Tables

## Supplementary Figures


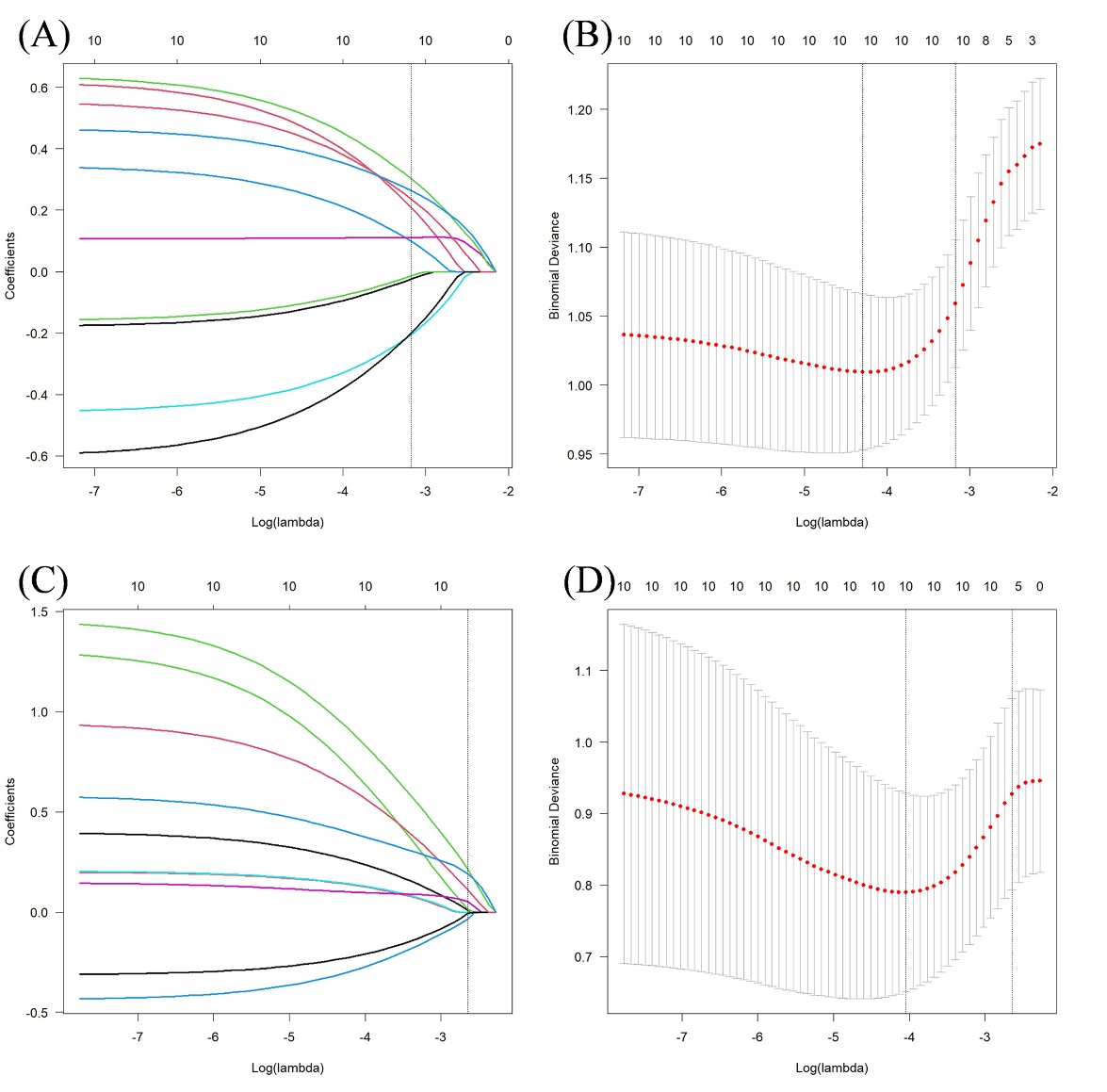


Supplementary Figure 1. (A) (B) Model 2 Lasso model in Task 1; (C) (D) Model 2 Lasso model in Task 2


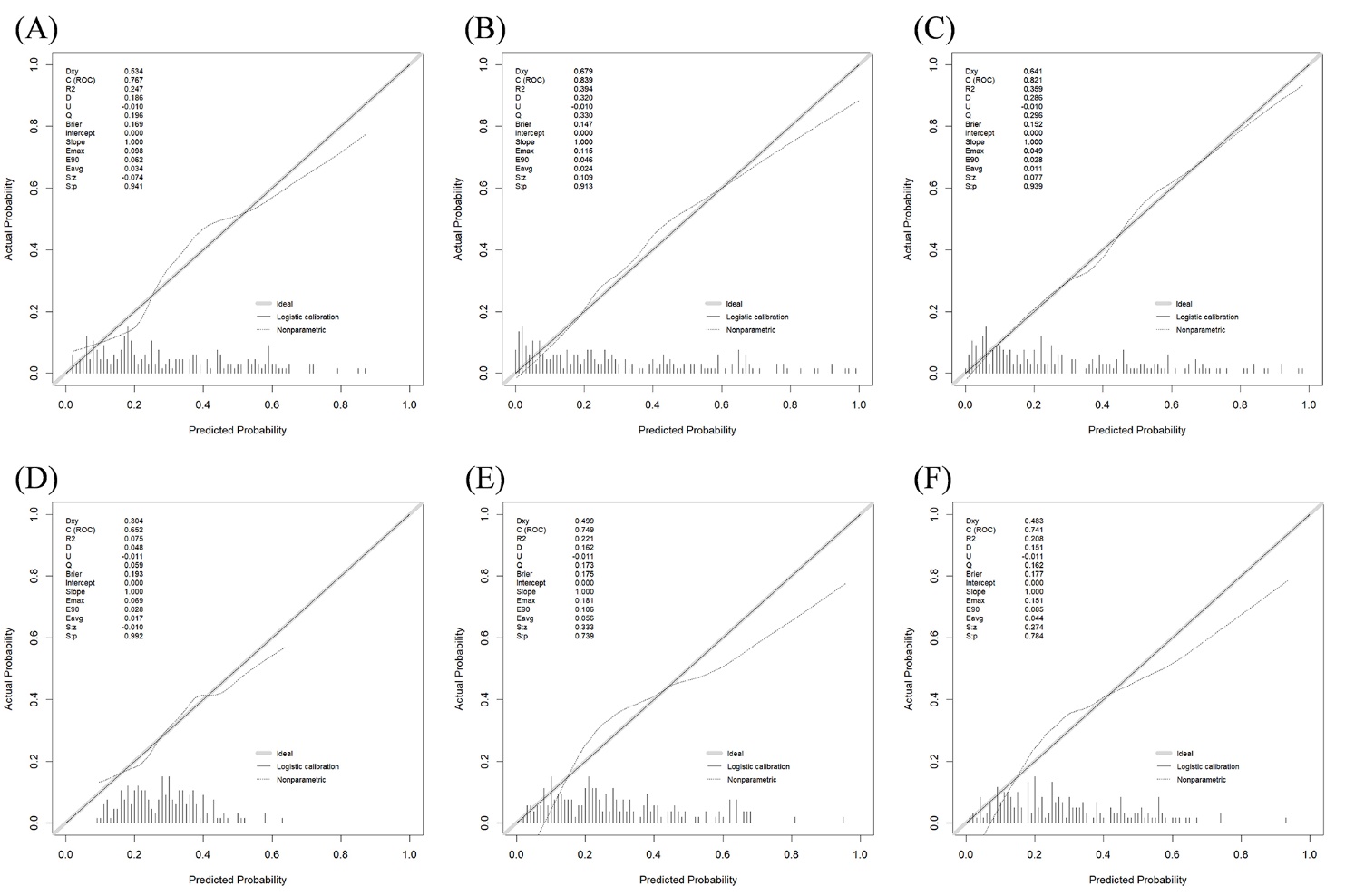


Supplementary Figure 2. Calibration curves in different datasets of three models in Task 1. (A) (B) (C) Calibration curves for the model 1, model 2, and model 3 in Data 1; (D) (E) (F) Calibration curves for the model 1, model 2, and model 3 in Data 2.
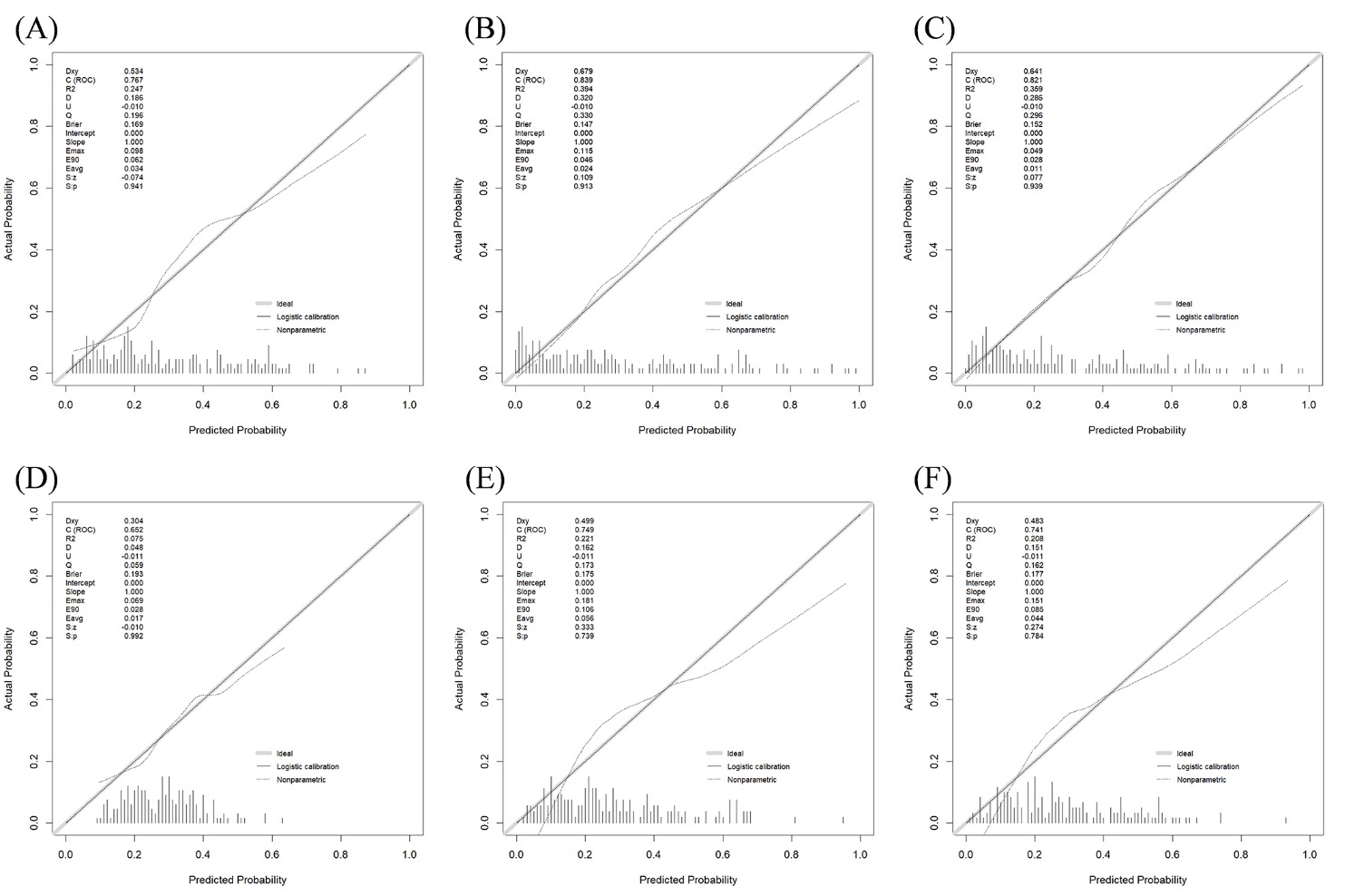


Supplementary Figure 3. Calibration curves in different datasets of three models in Task 2. (A) (B) (C) Calibration curves for the model 1, model 2, and model 3 in Data 1; (D) (E) (F) Calibration curves for the model 1, model 2, and model 3 in Data 2.


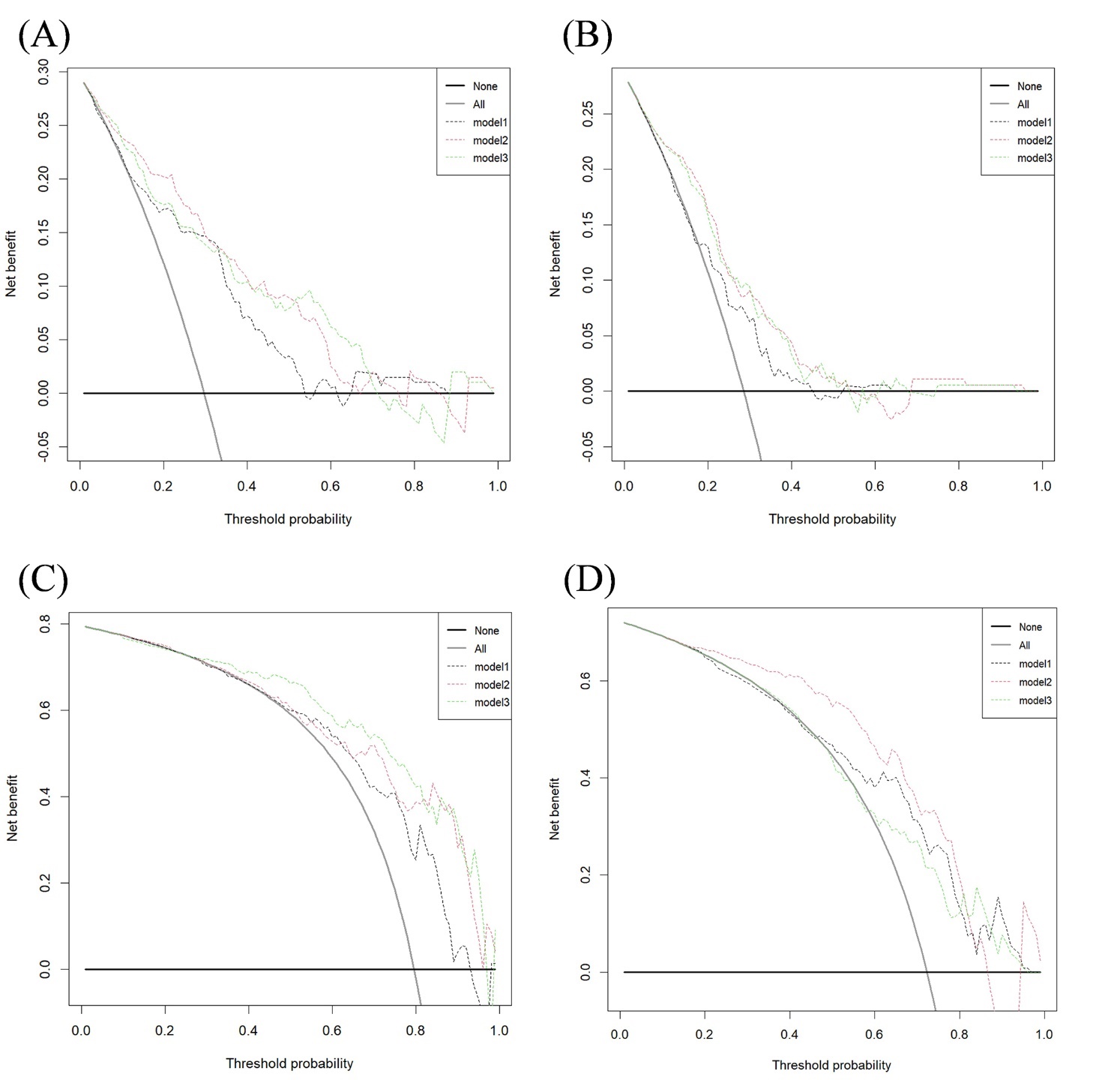


Supplementary Figure 4. (A) (B) DCA curves of model 2 for Task 1 in dataset 1 and dataset 2; (C) (D) DCA curves of model 2 for Task 2 in dataset 1 and dataset 2.

## Supplementary Tables

Supplementary Table 1. Comparison of data from the training set and the internal validation set in Task 1.

| Variables | Total (n = 202) | train (n = 141) | validation(n = 61) | p |
| --- | --- | --- | --- | --- |
| Age, Mean ± SD | 56.42 ± 10.85 | 56.79 ± 11.02 | 55.57 ± 10.5 | 0.459 |
| USsize, Median (Q1,Q3) | 2.1 (1.5, 2.6) | 2.1 (1.5, 2.6) | 2.1 (1.4, 2.6) | 0.739 |
| PATsize, Median (Q1,Q3) | 2.2 (1.5, 2.8) | 2 (1.5, 2.8) | 2.3 (1.5, 3) | 0.6 |
| USLN, n (%) |  |  |  | 0.57 |
| Negative | 152 (75) | 104 (74) | 48 (79) |  |
| Positive | 50 (25) | 37 (26) | 13 (21) |  |
| PatLN, n (%) |  |  |  | 0.887 |
| Negative | 126 (62) | 87 (62) | 39 (64) |  |
| Positive | 76 (38) | 54 (38) | 22 (36) |  |
| BIRADS, n (%) |  |  |  | 0.976 |
| 4A | 18 (9) | 13 (9) | 5 (8) |  |
| 4B | 49 (24) | 33 (23) | 16 (26) |  |
| 4C | 98 (49) | 69 (49) | 29 (48) |  |
| 5 | 37 (18) | 26 (18) | 11 (18) |  |
| SE, n (%) |  |  |  | 0.251 |
| 2 | 4 (2) | 3 (2) | 1 (2) |  |
| 3 | 27 (13) | 16 (11) | 11 (18) |  |
| 4 | 49 (24) | 31 (22) | 18 (30) |  |
| 5 | 122 (60) | 91 (65) | 31 (51) |  |
| SWVmean, Mean ± SD | 5.11 ± 1.65 | 5.16 ± 1.69 | 5 ± 1.57 | 0.509 |
| SWVmax, Median (Q1,Q3) | 6.32 (4.84, 8.26) | 6.36 (4.93, 8.26) | 6.05 (4.72, 8) | 0.583 |
| SWVmin, Median (Q1,Q3) | 3.86 (3.03, 4.71) | 3.84 (2.99, 4.75) | 3.89 (3.14, 4.4) | 0.885 |
| Convergence, n (%) |  |  |  | 0.117 |
| Negative | 114 (56) | 74 (52) | 40 (66) |  |
| Positive | 88 (44) | 67 (48) | 21 (34) |  |
| ER, n (%) |  |  |  | 0.021 |
| Negative | 44 (22) | 24 (17) | 20 (33) |  |
| Positive | 158 (78) | 117 (83) | 41 (67) |  |
| PR, n (%) |  |  |  | 0.132 |
| Negative | 69 (34) | 43 (30) | 26 (43) |  |
| Positive | 133 (66) | 98 (70) | 35 (57) |  |
| HER2, n (%) |  |  |  | 0.257 |
| Negative | 142 (70) | 103 (73) | 39 (64) |  |
| Positive | 60 (30) | 38 (27) | 22 (36) |  |
| KI67(20),n(%) |  |  |  | 0.847 |
| Low | 33 (16) | 24 (17) | 9 (15) |  |
| High | 169 (84) | 117 (83) | 52 (85) |  |
| TYPE, n (%) |  |  |  | 0.081 |
| HER-2+ | 16 (8) | 8 (6) | 8 (13) |  |
| LA | 24 (12) | 16 (11) | 8 (13) |  |
| LB- | 92 (46) | 72 (51) | 20 (33) |  |
| LB+ | 44 (22) | 30 (21) | 14 (23) |  |
| TN | 26 (13) | 15 (11) | 11 (18) |  |
| Radscore, Mean ± SD | -1.1 ± 0.73 | -1.11 ± 0.73 | -1.07 ± 0.73 | 0.767 |

Supplementary Table 2. Comparison of data from the training set and the internal validation set in Task 2.

| Variables | Total (n = 142) | train (n = 99) | validation (n = 43) | p |
| --- | --- | --- | --- | --- |
| Age, Mean ± SD | 56.69 ± 11.36 | 56.67 ± 11.69 | 56.74 ± 10.69 | 0.969 |
| USsize, Median (Q1,Q3) | 2 (1.4, 2.58) | 2 (1.45, 2.6) | 2.1 (1.4, 2.5) | 0.793 |
| PATsize, Median (Q1,Q3) | 2 (1.42, 2.6) | 2 (1.5, 2.75) | 2 (1.35, 2.55) | 0.452 |
| USLN, n (%) |  |  |  | 0.478 |
| Negative | 112 (79) | 76 (77) | 36 (84) |  |
| Positive | 30 (21) | 23 (23) | 7 (16) |  |
| PatLN, n (%) |  |  |  | 0.336 |
| Negative | 89 (63) | 59 (60) | 30 (70) |  |
| Positive | 53 (37) | 40 (40) | 13 (30) |  |
| BIRADS, n (%) |  |  |  | 0.116 |
| 4A | 13 (9) | 6 (6) | 7 (16) |  |
| 4B | 37 (26) | 30 (30) | 7 (16) |  |
| 4C | 63 (44) | 44 (44) | 19 (44) |  |
| 5 | 29 (20) | 19 (19) | 10 (23) |  |
| SE, n (%) |  |  |  | 0.896 |
| 2 | 3 (2) | 2 (2) | 1 (2) |  |
| 3 | 19 (13) | 14 (14) | 5 (12) |  |
| 4 | 31 (22) | 20 (20) | 11 (26) |  |
| 5 | 89 (63) | 63 (64) | 26 (60) |  |
| SWVmean, Mean ± SD | 5.07 ± 1.67 | 4.99 ± 1.67 | 5.25 ± 1.66 | 0.401 |
| SWVmax, Median (Q1,Q3) | 6.33 (4.86, 8.27) | 6.24 (5.14, 7.97) | 6.58 (4.51, 8.48) | 0.573 |
| SWVmin, Mean ± SD | 3.88 ± 1.23 | 3.88 ± 1.27 | 3.87 ± 1.16 | 0.956 |
| Convergence, n (%) |  |  |  | 0.61 |
| Negative | 73 (51) | 49 (49) | 24 (56) |  |
| Positive | 69 (49) | 50 (51) | 19 (44) |  |
| ER, n (%) |  |  |  | 0.88 |
| Negative | 27 (19) | 18 (18) | 9 (21) |  |
| Positive | 115 (81) | 81 (82) | 34 (79) |  |
| PR, n (%) |  |  |  | 0.702 |
| Negative | 35 (25) | 23 (23) | 12 (28) |  |
| Positive | 107 (75) | 76 (77) | 31 (72) |  |
| HER2, n (%) |  |  |  | 0.218 |
| Zero | 29 (20) | 17 (17) | 12 (28) |  |
| low | 113 (80) | 82 (83) | 31 (72) |  |
| KI6720, n (%) |  |  |  | 0.301 |
| Negative | 29 (20) | 23 (23) | 6 (14) |  |
| Positive | 113 (80) | 76 (77) | 37 (86) |  |
| TYPE, n (%) |  |  |  | 0.76 |
| LA | 24 (17) | 18 (18) | 6 (14) |  |
| LB- | 92 (65) | 64 (65) | 28 (65) |  |
| TN | 26 (18) | 17 (17) | 9 (21) |  |
